# Decay in chest compression quality due to fatigue is rare during prolonged advanced life support in a manikin model

**DOI:** 10.1186/1757-7241-19-46

**Published:** 2011-08-09

**Authors:** Conrad A Bjørshol, Kjetil Sunde, Helge Myklebust, Jörg Assmus, Eldar Søreide

**Affiliations:** 1 Department of Anaesthesiology and Intensive Care, Stavanger University Hospital, Stavanger, Norway; 2 Department of Anaesthesiology, Division of Critical Care, Oslo University Hospital, Oslo, Norway; 3 Laerdal Medical AS, Stavanger, Norway; 4 Centre for Clinical Research, Haukeland University Hospital, Bergen, Norway

**Keywords:** Advanced life support (ALS), cardiac arrest, cardiopulmonary resuscitation (CPR), fatigue, resuscitation, chest compression

## Abstract

**Background:**

The aim of this study was to measure chest compression decay during simulated advanced life support (ALS) in a cardiac arrest manikin model.

**Methods:**

19 paramedic teams, each consisting of three paramedics, performed ALS for 12 minutes with the same paramedic providing all chest compressions. The patient was a resuscitation manikin found in ventricular fibrillation (VF). The first shock terminated the VF and the patient remained in pulseless electrical activity (PEA) throughout the scenario. Average chest compression depth and rate was measured each minute for 12 minutes and divided into three groups based on chest compression quality; good (compression depth ≥ 40 mm, compression rate 100-120/minute for each minute of CPR), bad (initial compression depth < 40 mm, initial compression rate < 100 or > 120/minute) or decay (change from good to bad during the 12 minutes). Changes in no-flow ratio (NFR, defined as the time without chest compressions divided by the total time of the ALS scenario) over time was also measured.

**Results:**

Based on compression depth, 5 (26%), 9 (47%) and 5 (26%) were good, bad and with decay, respectively. Only one paramedic experienced decay within the first two minutes. Based on compression rate, 6 (32%), 6 (32%) and 7 (37%) were good, bad and with decay, respectively. NFR was 22% in both the 1-3 and 4-6 minute periods, respectively, but decreased to 14% in the 7-9 minute period (P = 0.002) and to 10% in the 10-12 minute period (P < 0.001).

**Conclusions:**

In this simulated cardiac arrest manikin study, only half of the providers achieved guideline recommended compression depth during prolonged ALS. Large inter-individual differences in chest compression quality were already present from the initiation of CPR. Chest compression decay and thereby fatigue within the first two minutes was rare.

## 1. Background

In cardiac arrest, good quality cardiopulmonary resuscitation (CPR) is essential for survival [1-3]. Together with early defibrillation [4,5], the quality of chest compressions is the main prerequisite for good outcome, especially chest compression depth [6] and avoidance of unnecessary hands-off intervals [4,5,7,8]. Current guidelines recommend changing the person providing chest compressions every two minutes [4,5]. Fatigue is supposed to be the main reason for this recommended practice [9-11], but the scientific evidence is limited. Since unnecessary changes in chest compressions may affect the overall quality of advanced life support (ALS) [12], we think this important topic deserves new attention.

In 1995, Hightower et al. described, in a manikin study with 11 study subjects, a decline in the quality of chest compressions over the first five minutes after initiating CPR [9]. The quality of the chest compressions was judged as inappropriate if the depth or hand placement was not within the recommendations. Subsequent manikin studies confirmed a decrease in chest compressions with adequate depth during the first few minutes of CPR [10,11,13,14]. However, based on the methodology used in these different studies it remains unclear whether this poor CPR performance is due to fatigue or other reasons. In contrast, two manikin studies have shown that CPR providers are able to perform chest compressions efficiently for 10 minutes while eliciting only moderate physiological stress [15], requiring just sub-anaerobic energy expenditure with no significant differences over the 10 minute study period [16]. In a previous manikin study we found no signs of chest compression decay during 10 minutes of single rescuer basic life support (BLS) by paramedics [17], but there was a huge inter-individual distribution in the quality of CPR. Similar data, with no obvious decline in chest compression quality over 5-10 minutes of BLS have also been described in lay people manikin studies [18,19], even when elderly people were tested [19].

Therefore, we decided to evaluate chest compression quality during a prolonged period of ALS in a manikin study with the same paramedic providing all chest compressions. We specifically wanted to focus on initial chest compression depth and if and when a decay in chest compression depth or rate occurred. Our hypothesis was that the degree of chest compression decay varied greatly between individual rescuers.

## 2. Methods

In a recently published randomised manikin study [20], 20 paramedic teams performed ALS under two different conditions; with and without socioemotional stress. The paramedics used had a median working experience of 8.5 years and participated in organised ALS training three to four times a year. The study was approved by the Regional Committee for Medical and Health Research Ethics. All participants signed an informed consent before entry.

The manikin was a modified Skillmeter Resusci Anne (Laerdal Medical, Stavanger, Norway) allowing simultaneous recording of ventilations and chest compressions. The manikin was found in ventricular fibrillation on the floor, and developed pulseless electrical activity (PEA) after the first shock. The manikin never achieved return of spontaneous circulation (ROSC). One paramedic in each paramedic team was randomised to perform all chest compressions.

**Figure 1:**
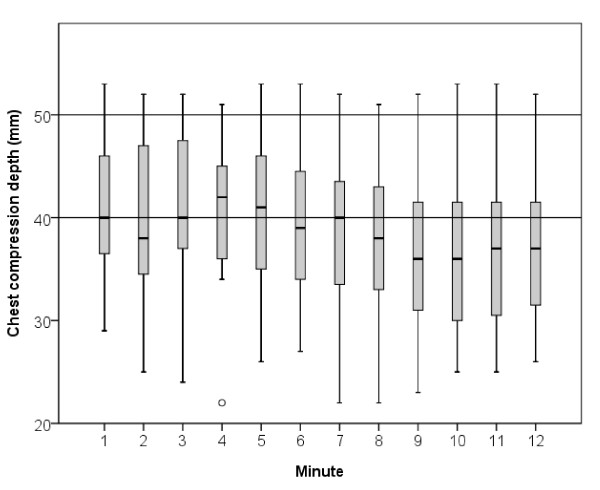
**Distribution of chest compression depth**. Boxplot showing the distribution of chest compression depths for each minute during twelve minutes of advanced life support on a manikin (n = 19). Centre line indicates median value, boxes indicate interquartile range and straight lines indicate maximum and minimum values. The circle denotes an outlier.

In the present study, we analysed specifically data from the condition where the paramedics were exposed to socioemotional stress, because this condition scored significantly higher on a subjective rating of realism (8.0 vs. 5.5, P < 0.001) [20]. The resuscitation attempts were discontinued at different times based on the time of intubation, but they all performed CPR for at least twelve minutes and continued the resuscitation attempt until they were told to stop. We therefore analysed the first twelve minutes of the resuscitation attempts. Starting by plotting the distribution of chest compression depth for each minute of ALS in a boxplot (Figure 1), this figure revealed, as demonstrated in our previous study [17], the great inter-individual variation in chest compression depth already evident in the first minute of ALS. Paramedics were thereafter described and grouped into different categories based on their initial chest compression depth. The resuscitation attempts were sorted into three different groups (good, bad and decay) based on the development of chest compression depth and rate over time. The following definitions were used, based on the recommendations from the 2005 guidelines [21,22]:

Good: CPR with average chest compression depth ≥ 40 mm for every minute during the 12 minute resuscitation attempt. Average chest compression rate 100-120 for every minute.

Bad: CPR with initial average chest compression depth < 40 mm. Chest compression rate < 100 or > 120 per minute at the start of the resuscitation attempt.

Decay: CPR with initial average chest compressions depths ≥ 40 mm which dropped below 40 mm. Chest compression rates 100-120 per minute that decreased to < 100 or increased to > 120 per minute.

The no-flow ratio (NFR) was defined as the time without chest compressions divided by the total time of the ALS scenario. The NFR was analysed in three minute periods because Norwegian ALS guidelines [23] recommend analysis of rhythm every three minutes, as opposed to international guidelines with their two minute periods [24,25]. The paramedics in the present study followed the Norwegian guidelines and have been thoroughly trained in these guidelines since 2006.

### Statistical analyses

We used SPSS version 17.0 (Chicago, IL, USA) for statistical analyses. Data are presented as mean values for each minute of ALS. We investigated the overall change in the NFR in the different three-minute periods using repeated measures ANOVA. Additionally we tested the difference between the first and each successive time interval pairwise using paired t tests. A P value of < 0.05 was regarded as significant. For the pairwise testing we had to take into account multiple testing effects, i.e. we adjusted the significance level using the Bonferroni correction. This leads to a significance level of 0.017 (3 pairwise tests).

## 3. Results

Altogether 20 paramedic teams completed the study. One registration failed due to software failure. Hence, 19 ALS resuscitations were available for this chest compression quality analysis. In each resuscitation attempt, the same paramedic performed all the chest compressions, and 68% of the chest compression providers were male.

**Figure 2:**
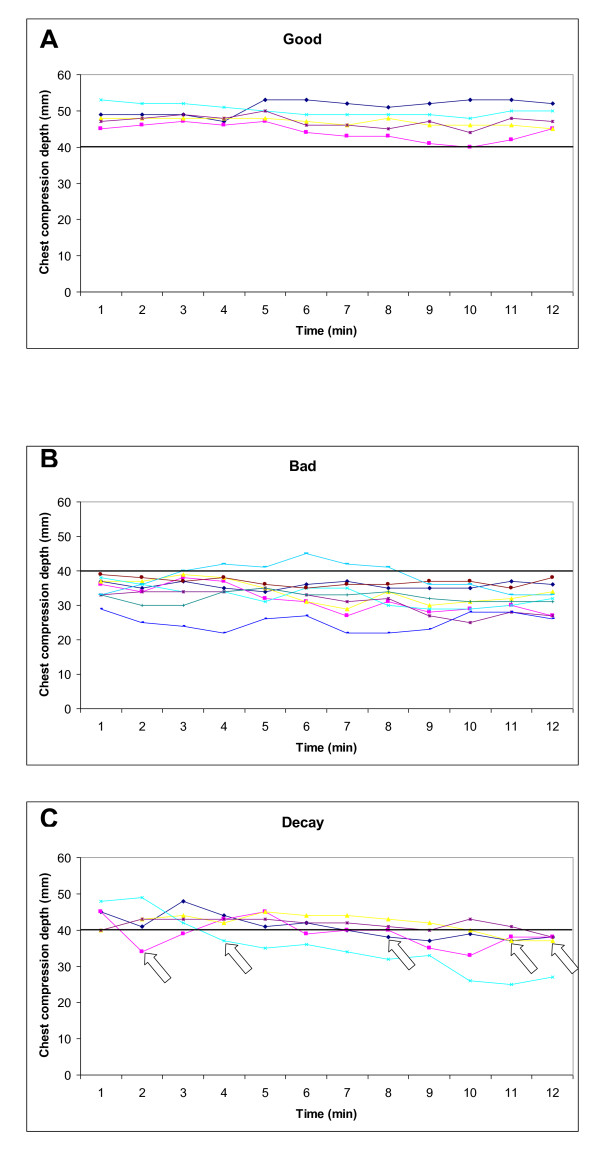
**Development of chest compression depth**. Development of chest compression depth for each of 19 resuscitation attempts, the good are illustrated in A (5/19, 26%), the bad in B (9/19, 47%) and those with decay in C (5/19, 26%). Arrows indicate when each paramedic first developed decay in chest compression depth to < 40 mm. See text for definition of groups.

Based on chest compression depth, 26% (5/19) and 47% (9/19) of the ALS resuscitations were classified as good and bad throughout the 12 minute scenario, respectively. In these cases no signs of decay or major changes occurred (Figure 2), except for one among the bad, where sufficient chest compression depth was achieved between 3 and 8 minutes (Figure 2B). In 26% (5/19) of the cases, decay in chest compression depth was present. Of these five cases, only one paramedic displayed chest compression decay to below 40 mm within the first two minutes, the remainder after 4, 8, 11 and 12 minutes (Figure 2C).

**Figure 3:**
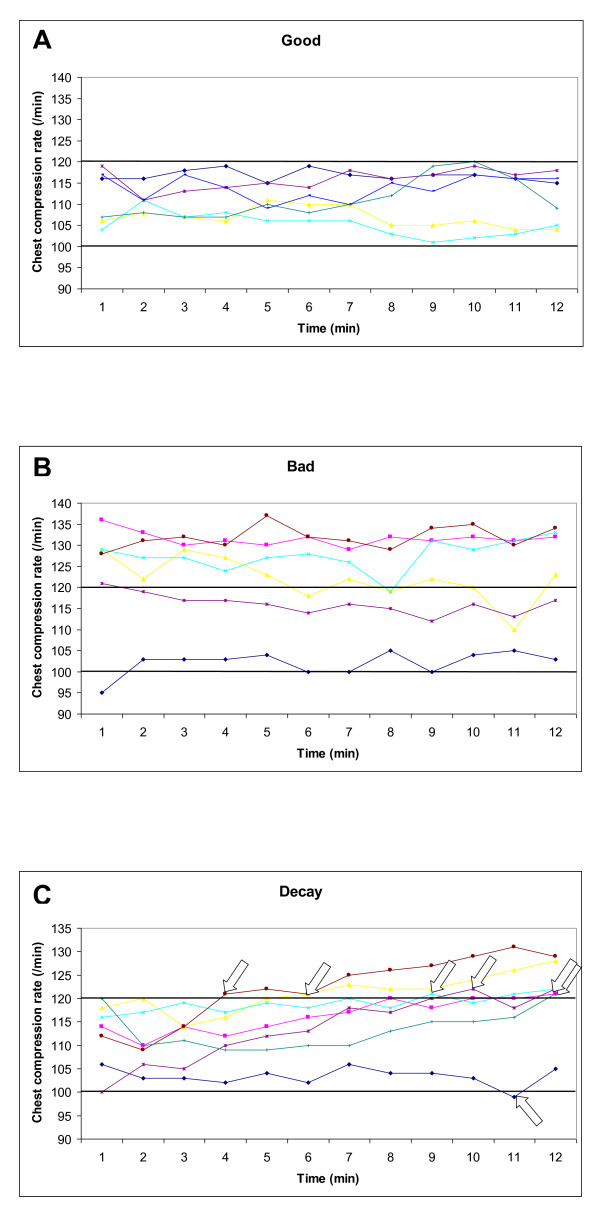
**Development of chest compression rate**. Development of chest compression rate for each of 19 resuscitation attempts, the good are illustrated in A (6/19, 32%), the bad in B (6/19, 32%) and those with decay in C (7/19, 37%). Arrows indicate when each paramedic developed decay in chest compression rate to < 100 or > 120 per minute. See text for definition of groups.

Based on chest compression rate, 32% (6/19) of the resuscitation attempts were scored as good and 32% (6/19) as bad. Among the bad, two achieved correct rate after the first minute. Decay was present in 37% (7/19) of the cases, and only one was evident in the first five minutes of ALS (Figure 3).

**Figure 4:**
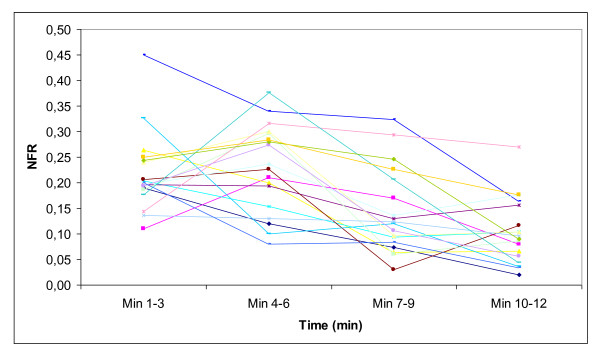
**Development of no-flow ratio**. Development of no-flow ratio measured in three minute periods for all 19 resuscitation attempts.

Average NFR for the 19 paramedics was 17%, with a range from 10 to 32%, and NFR changed significantly over time (P < 0.001). NFR remained unchanged at 22% in the 1-3 minute and 4-6 minute periods, but decreased to 14% from the 1-3 minute period to the 7-9 minute period (P = 0.002) and further to 10% from the 1-3 minute period to the 10-12 minute period (P < 0.001) (Figure 4).

## 4. Discussion

In this manikin study, where each paramedic performed 12 minutes of chest compressions in a realistic ALS scenario, we demonstrated that huge inter-individual differences in chest compression depth and rate exist. This is present already from the initiation of ALS. Decay due to fatigue seems to be a less frequent problem, as only five and six out of 19 paramedics developed decay in chest compression depth and rate, respectively. Noteworthy, only one paramedic showed decay in chest compression depth within the initial two minutes, and only one showed decay in compression rate within the initial five minutes.

A manikin study by Hightower et al. from 1995, where 11 nursing assistants performed chest compressions for five minutes [9], described a significant and steady decline in the percentage of correct compressions already evident in the second minute. The authors speculate that fatigue might be the reason for this compression quality decay without specifying whether the incorrect compressions were due to incomplete compression depth or wrong hand placement. Later manikin studies showed similar results with a decline in chest compression depth after the initial minutes of the CPR attempt [10,11,13,14,26]. A clinical study on in-hospital cardiac arrested patients [27] described a decay in chest compression depth that was statistically significant after only 90 seconds. However, no correction was made for different surfaces on which the patients were located. These previous studies all conclude that decay in mean chest compression depth is evident after a very short period of time. Importantly, their data analyses do not take into account the huge inter-individual differences among the CPR providers that will influence the results. We have in a previous BLS manikin study [17], as in the present ALS manikin study, documented that these inter-individual differences are present already from the initiation of CPR. Thus, it was necessary to analyse the data by sorting the individuals into different groups based on their initial chest compression quality, instead of calculating mean values for a large group of individuals.

In the 2010 guidelines optimal chest compression quality is even more emphasized than previously, and a chest compression depth of at least 50 mm is recommended [4,5]. Although our paramedics were trained in the previous guidelines recommending a compression depth of 40-50 mm, it is a cause of concern that 47% in the present study had chest compression depths of less than 40 mm already from the initiation of CPR. As seen in Figure 2B, this is not a result of fatigue or chest compression decay, but an inappropriate chest compression depth already from initiation of CPR. There are several potential reasons for this deviation from guidelines; insufficient muscular power, lack of sufficient body weight, as weight previously has been correlated with compression depth [28], an inaccuracy of chest compression depth because no feedback was available, or a fear of causing serious patient injury [29]. In a questionnaire among Norwegian and UK paramedics, Ødegaard et al. reported that many paramedics had concerns causing serious patient injuries if they compressed to the guidelines' depth [29]. Thus, it is very relevant to highlight chest compressions quality, especially compression depth, in ALS training and practise in the future. The fear of causing patient injuries must be overcome.

More positive, all paramedics had compression rates above 100 per minute for the majority of the resuscitation attempts. This is important as higher compression rates increase cardiac output resulting in increased myocardial and cerebral blood flow [30,31] and improved short-term survival in humans [32]. Decay in chest compression rate over time was rare and only evident in one paramedic within the first five minutes. 26% initiated CPR with chest compression rates above 120 per minute. This is unfavourable as coronary perfusion is reduced at rates over 120-130 per minute [31], thereby reducing the probability of successful resuscitation [33]. A metronome [34,35] or real time feedback [36] could improve the chest compression rate.

NFR did not increase over time in our study but actually declined, even though the same rescuer provided all the chest compressions for as long as 12 minutes. One likely explanation for this positive, continuous decrease in NFR over time is that the patient in our scenario developed PEA after the first shock, and hence there was no further need for charging the defibrillator and shocking the patient. On the other hand, an organised ECG rhythm necessitates pulse checks to differentiate PEA from ROSC in the absence of end-tidal CO_2_-measurement (ETCO_2_), and hence further increases the NFR. Further, as the patient was intubated after about five minutes [20], this could have contributed to the reduced NFR as this allows for simultaneous ventilations and continuous chest compressions [37]. A clinical observation study has also shown no increase in NFR over time [38]. Our paramedics had a NFR of 17% in the 12 minute study period which is comparable to recent clinical observation studies [39,40], and far better than data from the recent US ROC trials with NFR between 34 and 46% [36,41].

Importantly, based on our findings it seems unwarranted to recommend changing the person providing chest compressions every two minutes during ALS as recommended in the new resuscitation guidelines. It has been shown that provider switches account for at least 40% of NFR during CPR [12], and this can be reduced by avoiding unnecessary switches. Instead of changing chest compression provider frequently, we recommend more attention on optimising chest compression quality already from the initiation of CPR, and that the chest compression quality should be monitored continuously with CPR feedback devices or capnography during ALS. CPR feedback devices have been shown to improve the quality of CPR, including chest compression depth and ROSC rate, but still have not led to increased long-term survival [36,42]. Capnography, with ETCO_2 _measurements, predicts cardiac output [43] and is correlated with both ROSC and survival [44]. However, more studies are needed to show if CPR feedback devices or capnography can assist in finding the optimal time point for switching the provider of chest compressions.

There are limitations to this study. As it was a simulation manikin study, we do not know whether the quality of chest compression is compromised more or less in real cardiac arrest situations. It has been shown that paramedics are physically capable of compressing to guideline depth for 5 minutes even on a manikin with chest stiffness mimicking the upper eighth of chest stiffnesses in a patient population [29]. The manikin in our study does not represent the large variation in stiffness and damping found in human chests during CPR [45,46]. Further, our study included paramedics with a median experience of 8.5 years and frequent refresher training in ALS. We do not know if chest compression decay or chest compression quality in general is different for less experienced paramedics and other health care providers. As this is the first study to explore chest compression decay by sorting individuals based on compression quality, a power analysis was not performed and hence we cannot rule out that our results are caused by insufficient power. Finally, we followed the recommendations from the Norwegian 2005 guidelines in the present study [23], with 4 cm of chest compression depth regarded as good. We might speculate that the 5 cm recommendation from 2010 would have caused more decay and fatigue, especially if every paramedic initially compressed to the guidelines depth. Further studies are indeed warranted.

## 5. Conclusion

In this simulated cardiac arrest manikin study, only half of the providers achieved guideline recommended compression depth during prolonged ALS. Large inter-individual differences in chest compression quality were already present from the initiation of CPR. Chest compression decay and thereby fatigue within the first two minutes was rare.

## 6. Competing interests

CAB has a part-time employment as facilitator at Stavanger Acute Medicine Foundation for Education and Research (SAFER). ES is medical director at SAFER. CAB and ES have received financial support from the Laerdal Foundation for Acute Medicine. HM is an employee of Laerdal Medical. KS and JA have no competing interests.

## 7. Authors' contributions

CAB participated in study design, running the simulations, statistical analyses and manuscript writing, KS and ES in study design and manuscript writing, HM in study design, running simulations and manuscript writing, and JA in statistical analyses and manuscript writing. All authors read and approved the final manuscript.

## References

[B1] Gallagher EJ Lombardi G Gennis P Effectiveness of bystander cardiopulmonary resuscitation and survival following out-of-hospital cardiac arrest JAMA 1995 274 1922 1925 10.1001/jama.274.24.1922 8568985

[B2] Van Hoeyweghen RJ Bossaert LL Mullie A Calle P Martens P Buylaert WA Delooz H Quality and efficiency of bystander CPR. Belgian Cerebral Resuscitation Study Group Resuscitation 1993 26 47 52 10.1016/0300-9572(93)90162-J 8210731

[B3] Wik L Steen PA Bircher NG Quality of bystander cardiopulmonary resuscitation influences outcome after prehospital cardiac arrest Resuscitation 1994 28 195 203 10.1016/0300-9572(94)90064-7 7740189

[B4] Koster RW Baubin MA Bossaert LL Caballero A Cassan P Castren M Granja C Handley AJ Monsieurs KG Perkins GD Raffay V Sandroni C European Resuscitation Council Guidelines for Resuscitation 2010 Section 2. Adult basic life support and use of automated external defibrillators Resuscitation 2010 81 1277 1292 10.1016/j.resuscitation.2010.08.009 20956051 PMC7116923

[B5] Berg RA Hemphill R Abella BS Aufderheide TP Cave DM Hazinski MF Lerner EB Rea TD Sayre MR Swor RA Part 5: adult basic life support: 2010 American Heart Association Guidelines for Cardiopulmonary Resuscitation and Emergency Cardiovascular Care Circulation 2010 122 S685 705 10.1161/CIRCULATIONAHA.110.970939 20956221

[B6] Kramer-Johansen J Myklebust H Wik L Fellows B Svensson L Sørebø H Steen PA Quality of out-of-hospital cardiopulmonary resuscitation with real time automated feedback: a prospective interventional study Resuscitation 2006 71 283 292 10.1016/j.resuscitation.2006.05.011 17070980

[B7] Eftestøl T Sunde K Steen PA Effects of interrupting precordial compressions on the calculated probability of defibrillation success during out-of-hospital cardiac arrest Circulation 2002 105 2270 2273 10.1161/01.CIR.0000016362.42586.FE 12010909

[B8] Edelson DP Abella BS Kramer-Johansen J Wik L Myklebust H Barry AM Merchant RM Hoek TL Steen PA Becker LB Effects of compression depth and pre-shock pauses predict defibrillation failure during cardiac arrest Resuscitation 2006 71 137 145 10.1016/j.resuscitation.2006.04.008 16982127

[B9] Hightower D Thomas SH Stone CK Dunn K March JA Decay in quality of closed-chest compressions over time Ann Emerg Med 1995 26 300 303 10.1016/S0196-0644(95)70076-5 7661418

[B10] Ochoa FJ Ramalle-Gomara E Lisa V Saralegui I The effect of rescuer fatigue on the quality of chest compressions Resuscitation 1998 37 149 152 10.1016/S0300-9572(98)00057-4 9715774

[B11] Ashton A McCluskey A Gwinnutt CL Keenan AM Effect of rescuer fatigue on performance of continuous external chest compressions over 3 min Resuscitation 2002 55 151 155 10.1016/S0300-9572(02)00168-5 12413752

[B12] Sutton RM Maltese MR Niles D French B Nishisaki A Arbogast KB Donoghue A Berg RA Helfaer MA Nadkarni V Quantitative analysis of chest compression interruptions during in-hospital resuscitation of older children and adolescents Resuscitation 2009 80 1259 1263 10.1016/j.resuscitation.2009.08.009 19733427

[B13] Greingor JL Quality of cardiac massage with ratio compression-ventilation 5/1 and 15/2 Resuscitation 2002 55 263 267 10.1016/S0300-9572(02)00237-X 12458063

[B14] Jäntti H Silfvast T Turpeinen A Kiviniemi V Uusaro A Quality of cardiopulmonary resuscitation on manikins: on the floor and in the bed Acta Anaesthesiol Scand 2009 53 1131 1137 10.1111/j.1399-6576.2009.01966.x 19388894

[B15] Miles DS Underwood PD Jr Nolan DJ Frey MA Gotshall RW Metabolic, hemodynamic, and respiratory responses to performing cardiopulmonary resuscitation Can J Appl Sport Sci 1984 9 141 147 6488434

[B16] Shultz JJ Mianulli MJ Gisch TM Coffeen PR Haidet GC Lurie KG Comparison of exertion required to perform standard and active compression-decompression cardiopulmonary resuscitation Resuscitation 1995 29 23 31 10.1016/0300-9572(94)00812-T 7784719

[B17] Bjørshol CA Søreide E Torsteinbø TH Lexow K Nilsen OB Sunde K Quality of chest compressions during 10 min of single-rescuer basic life support with different compression: ventilation ratios in a manikin model Resuscitation 2008 77 95 100 10.1016/j.resuscitation.2007.11.009 18207627

[B18] Ødegaard S Saether E Steen PA Wik L Quality of lay person CPR performance with compression: ventilation ratios 15:2, 30:2 or continuous chest compressions without ventilations on manikins Resuscitation 2006 71 335 340 10.1016/j.resuscitation.2006.05.012 17069958

[B19] Neset A Birkenes TS Myklebust H Mykletun RJ ødegaard S Kramer-Johansen J A randomized trial of the capability of elderly lay persons to perform chest compression only CPR versus standard 30:2 CPR Resuscitation 2010 81 887 892 10.1016/j.resuscitation.2010.03.028 20418006

[B20] Bjørshol CA Myklebust H Nilsen KL Hoff T Bjørkli C Illguth E Soreide E Sunde K Effect of socioemotional stress on the quality of cardiopulmonary resuscitation during advanced life support in a randomized manikin study Crit Care Med 2011 39 300 304 10.1097/CCM.0b013e3181ffe100 21076285

[B21] Handley AJ Koster R Monsieurs K Perkins GD Davies S Bossaert L European Resuscitation Council guidelines for resuscitation 2005. Section 2. Adult basic life support and use of automated external defibrillators Resuscitation 2005 67 Suppl 1 S7 23 16321717 10.1016/j.resuscitation.2005.10.007

[B22] 2005 American Heart Association Guidelines for Cardiopulmonary Resuscitation and Emergency Cardiovascular Care Circulation 2005 112 IV1 203 16314375 10.1161/CIRCULATIONAHA.105.166550

[B23] Lexow K Sunde K Why Norwegian 2005 guidelines differs slightly from the ERC guidelines Resuscitation 2007 72 490 492 10.1016/j.resuscitation.2006.07.018 17161898

[B24] Deakin CD Nolan JP Soar J Sunde K Koster RW Smith GB Perkins GD European Resuscitation Council Guidelines for Resuscitation 2010 Section 4. Adult advanced life support Resuscitation 2010 81 1305 1352 10.1016/j.resuscitation.2010.08.017 20956049

[B25] Neumar RW Otto CW Link MS Kronick SL Shuster M Callaway CW Kudenchuk PJ Ornato JP McNally B Silvers SM Passman RS White RD Hess EP Tang W Davis D Sinz E Morrison LJ Part 8: adult advanced cardiovascular life support: 2010 American Heart Association Guidelines for Cardiopulmonary Resuscitation and Emergency Cardiovascular Care Circulation 2010 122 S729 767 10.1161/CIRCULATIONAHA.110.970988 20956224

[B26] Heidenreich JW Berg RA Higdon TA Ewy GA Kern KB Sanders AB Rescuer fatigue: standard versus continuous chest-compression cardiopulmonary resuscitation Acad Emerg Med 2006 13 1020 1026 10.1111/j.1553-2712.2006.tb00272.x 17015418

[B27] Sugerman NT Edelson DP Leary M Weidman EK Herzberg DL Vanden Hoek TL Becker LB Abella BS Rescuer fatigue during actual in-hospital cardiopulmonary resuscitation with audiovisual feedback: A prospective multicenter study Resuscitation 2009 80 981 984 10.1016/j.resuscitation.2009.06.002 19581036 PMC2746377

[B28] Larsen PD Perrin K Galletly DC Patterns of external chest compression Resuscitation 2002 53 281 287 10.1016/S0300-9572(02)00026-6 12062844

[B29] Ødegaard S Kramer-Johansen J Bromley A Myklebust H Nysaether J Wik L Steen PA Chest compressions by ambulance personnel on chests with variable stiffness: Abilities and attitudes Resuscitation 2007 74 127 134 10.1016/j.resuscitation.2006.12.006 17368692

[B30] Sunde K Wik L Naess PA Grund F Nicolaysen G Steen PA Improved haemodynamics with increased compression-decompression rates during ACD-CPR in pigs Resuscitation 1998 39 197 205 10.1016/S0300-9572(98)00139-7 10078810

[B31] Wolfe JA Maier GW Newton JR Jr Glower DD Tyson GS Jr Spratt JA Rankin JS Olsen CO Physiologic determinants of coronary blood flow during external cardiac massage The Journal of thoracic and cardiovascular surgery 1988 95 523 532 3343860

[B32] Abella BS Sandbo N Vassilatos P Alvarado JP O'Hearn N Wigder HN Hoffman P Tynus K Vanden Hoek TL Becker LB Chest compression rates during cardiopulmonary resuscitation are suboptimal: a prospective study during in-hospital cardiac arrest Circulation 2005 111 428 434 10.1161/01.CIR.0000153811.84257.59 15687130

[B33] Paradis NA Martin GB Rivers EP Goetting MG Appleton TJ Feingold M Nowak RM Coronary perfusion pressure and the return of spontaneous circulation in human cardiopulmonary resuscitation JAMA 1990 263 1106 1113 10.1001/jama.263.8.1106 2386557

[B34] Jäntti H Silfvast T Turpeinen A Kiviniemi V Uusaro A Influence of chest compression rate guidance on the quality of cardiopulmonary resuscitation performed on manikins Resuscitation 2009 80 453 457 10.1016/j.resuscitation.2009.01.001 19203821

[B35] Milander MM Hiscok PS Sanders AB Kern KB Berg RA Ewy GA Chest compression and ventilation rates during cardiopulmonary resuscitation: the effects of audible tone guidance Acad Emerg Med 1995 2 708 713 10.1111/j.1553-2712.1995.tb03622.x 7584749

[B36] Hostler D Everson-Stewart S Rea TD Stiell IG Callaway CW Kudenchuk PJ Sears GK Emerson SS Nichol G Effect of real-time feedback during cardiopulmonary resuscitation outside hospital: prospective, cluster-randomised trial Bmj 2011 342 d512 10.1136/bmj.d512 21296838 PMC3033623

[B37] Kramer-Johansen J Wik L Steen PA Advanced cardiac life support before and after tracheal intubation--direct measurements of quality Resuscitation 2006 68 61 69 10.1016/j.resuscitation.2005.05.020 16325329

[B38] Wik L Kramer-Johansen J Myklebust H Sørebø H Svensson L Fellows B Steen PA Quality of cardiopulmonary resuscitation during out-of-hospital cardiac arrest JAMA 2005 293 299 304 10.1001/jama.293.3.299 15657322

[B39] Olasveengen TM Wik L Kramer-Johansen J Sunde K Pytte M Steen PA Is CPR quality improving? A retrospective study of out-of-hospital cardiac arrest Resuscitation 2007 75 260 266 10.1016/j.resuscitation.2007.04.016 17560005

[B40] Olasveengen TM Sunde K Brunborg C Thowsen J Steen PA Wik L Intravenous drug administration during out-of-hospital cardiac arrest: a randomized trial JAMA 2009 302 2222 2229 10.1001/jama.2009.1729 19934423

[B41] Christenson J Andrusiek D Everson-Stewart S Kudenchuk P Hostler D Powell J Callaway CW Bishop D Vaillancourt C Davis D Aufderheide TP Idris A Stouffer JA Stiell I Berg R Chest compression fraction determines survival in patients with out-of-hospital ventricular fibrillation Circulation 2009 120 1241 1247 10.1161/CIRCULATIONAHA.109.852202 19752324 PMC2795631

[B42] Edelson DP Robertson-Dick BJ Yuen TC Eilevstjønn J Walsh D Bareis CJ Vanden Hoek TL Abella BS Safety and efficacy of defibrillator charging during ongoing chest compressions: a multi-center study Resuscitation 2010 81 1521 1526 10.1016/j.resuscitation.2010.07.014 20807672 PMC3768293

[B43] Weil MH Bisera J Trevino RP Rackow EC Cardiac output and end-tidal carbon dioxide Crit Care Med 1985 13 907 909 10.1097/00003246-198511000-00011 3931979

[B44] Grmec S Klemen P Does the end-tidal carbon dioxide (EtCO2) concentration have prognostic value during out-of-hospital cardiac arrest? Eur J Emerg Med 2001 8 263 269 10.1097/00063110-200112000-00003 11785591

[B45] Nysaether JB Dorph E Rafoss I Steen PA Manikins with human-like chest properties--a new tool for chest compression research IEEE transactions on bio-medical engineering 2008 55 2643 2650 18990635 10.1109/TBME.2008.2001289

[B46] Tomlinson AE Nysaether J Kramer-Johansen J Steen PA Dorph E Compression force-depth relationship during out-of-hospital cardiopulmonary resuscitation Resuscitation 2007 72 364 370 10.1016/j.resuscitation.2006.07.017 17141936

